# Assessment of the Effects of Access Count in Percutaneous Nephrolithotomy on Renal Functions by Technetium-99m-Dimercaptosuccinic Acid Scintigraphy

**DOI:** 10.1155/2013/827121

**Published:** 2013-05-08

**Authors:** Abdullah Demirtaş, Mehmet Caniklioğlu, Mustafa Kula, Mustafa Sofikerim, Emre Can Akınsal, Mehmet Ali Ergül, Numan Baydilli, Oğuz Ekemekçioğlu

**Affiliations:** ^1^Department of Urology, Erciyes University Faculty of Medicine, 38039 Kayseri, Turkey; ^2^Department of Nuclear Medicine, Erciyes University Faculty of Medicine, 38039 Kayseri, Turkey; ^3^Department of Urology, Acibadem University Faculty of Medicine, 34848 Istanbul, Turkey

## Abstract

*Objective*. To determine the effects of percutaneous nephrolithotomy on renal functions by using DMSA scintigraphy while considering access counts. *Material and Methods*. A total of 37 patients who had undergone percutaneous nephrolithotomy were included. Preoperative DMSA scans were performed a day before the surgery, whereas postoperative scans were randomized by evaluating them before (*n* = 25) and after (*n* = 12) the 6th postoperative month. Twenty-six of 37 cases underwent percutaneous nephrolithotomy with a single access site and 11 with multiple access sites. *Results*. There were no significant changes of total renal functions in the whole study group (*P* = 0.054). In the single access group, total functions were significantly elevated (*P* = 0.03) In the multiple access group, while treated site functions were significantly decreased (*P* = 0.01), total functions did not change significantly (*P* = 0.42). There was an insignificant decrease in those evaluated before the 6th postoperative month (*P* = 0.27) and an insignificant increase in the others (*P* = 0.11). *Conclusion*. We could not find a superiority of single access over multiple accesses. There is a temporary functional loss in the treated site.

## 1. Introduction

Urinary stone disease (USD) is an important and frequently seen health problem. The main problem is recurrence. In human beings it can recur at a rate of approximately 50% in five years [1]. Therefore, USD still does not have a definitive treatment. Meticulous clearance of stones, minimal morbidity, maximal nephron sparing, and slower recurrence rate should be aimed at during treatment. In this context extracorporeal shockwave lithotripsy (ESWL) and minimally invasive surgeries are generally performed instead of open surgery nowadays [2]. Although there are numerous reports about the percutaneous nephrolithotomy (PCNL) technique and results, its effects on renal functions are not well known quantitatively [3]. 99m-Technetium-Dimercaptosuccinic Acid (DMSA) scintigraphy is very helpful for assessing parenchymal function after a surgical intervention [4]. 

In this study we aimed to determine the effects of PCNL on renal functions by using DMSA scintigraphy while considering tract and dilatation counts. In addition, we discussed the effect of surgical trauma on the local surgical area (dilatation site on the kidney).

## 2. Material and Methods

This study was approved by the local ethics committee (ID: 07/45). The informed consent was signed by all patients. Total 37 patients who had undergone PCNL were included between June 2007 and June 2009. All patients were evaluated routinely with physical examination, complete blood count (CBC), blood urine nitrogen (BUN), creatinine levels, and urinalysis. None of the patients had experienced pyelonephritis, and none had a solitary kidney, renal ectopy, or history of any other urinary abnormality. At least one of the following techniques, namely, computed tomography (CT), intravenous urography, or ultrasonography, was preferred routinely before surgery in order to visualize the urinary system. Unilateral PCNL was performed in all patients. Postoperative CBC and BUN creatinine levels were repeated.

**Figure 1:**
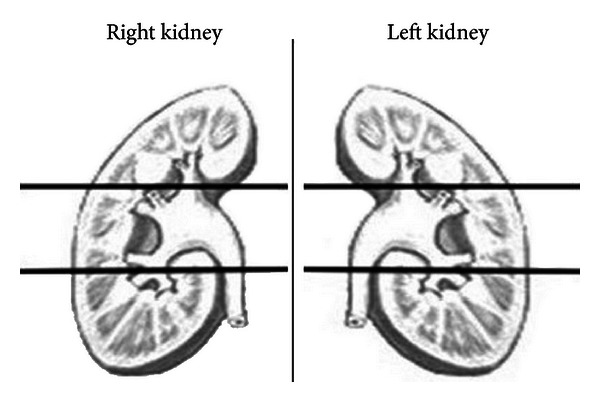
The three paired poles of both kidneys for DMSA scan evaluation.

Preoperative DMSA scans were performed a day before the surgery, whereas postoperative scans were randomized to indicate early and late term. The aim of randomization was to determine an optimal time for assessing patients. A DMSA scan was read by using a technique that divides both kidneys into three paired poles. In addition, the uptake of all opposite poles was measured together and calculated as a percentage value separately (Figure 1); for example, the two upper poles' uptakes were measured together as if they were renal units, and each pole's own portion in this total uptake was declared separately as a percentage. How the differential functions were changed between the sides undergoing PCNL and the opposite sides as well the changes before and 6 months after surgery are manifested by using these parameters.

Twenty-six of 37 cases underwent PCNL with a single access site (70.3%) and 11 separately with multiple accesses (29.7%). When each of the poles of a kidney was considered as a surgical unit, there were 51 units. In this manner the functional change of a unit would show the surgical trauma inflicted on the poles by PCNL access.

## 3. Results

DMSA scintigraphies and serum biochemical parameters were compared preoperatively and postoperatively. The postoperative total scintigraphic functions showed an insignificant elevation when compared to preoperative functions in the entire group (*P* = 0.054). Creatinine levels were elevated significantly (*P* = 0.03) (Table 1).

The treated sides' differential functions decreased significantly after the procedure when compared to preoperative levels (*P* = 0.003). As for the nontreated sides' functions, they increased significantly (*P* = 0.014). When considered according to evaluation time, in those which were evaluated before the postoperative 6th month (early term), the treated side decreased significantly and the other side increased significantly. After the 6th month (late term), the treated sides' functions significant decrease continued, and the other side had no significant change (Table 2).

Twenty-six of 37 cases underwent PCNL with a single access (70.3%) and 11 with multiple access sites (29.7%). When each of the poles of a kidney was admitted as a surgical unit separately, there were 51 accessed units. We examined how access affects the renal parenchymal function in the dilatation site. After evaluation there was a significant decrease at the access site (*P* = 0.041) (Table 3). This change was not related to multiple access sites or demographical or clinical parameters.

Changes at the nonaccessed poles of the treated sides were researched. The nonaccessed poles' count was 60. They were compared according to their functions before and after surgery, and there was no significant change (*P* = 0.43).

The 51 accessed units (that were mentioned above) were divided into two as single and multiple accessed groups. Also they were examined according to the total function and treated side and nontreated side function values, by comparing the before and after surgery values. In the single access group (*n* = 26) total functions and nontreated side functions were significantly elevated (resp., *P* = 0.035 and *P* = 0.022). On the other hand, the treated side showed no significant change (*P* = 0.067). In the multiple access group (*n* = 11) while the treated sides' functions were significantly decreased (*P* = 0.01) the other parameters did not change significantly. These groups were also examined according to the biochemical parameters. The creatinine levels of the single access group were significantly increased (*P* = 0.003), while the multiple access group did not show a significant change in BUN or creatinine levels (Table 4).

The postoperative scintigraphic assessment was performed before or after the 6th postoperative month. Therefore, the group evaluated before the 6th postoperative month is called the early term group and the other as the late term group. However, the early period of surgical trauma was evaluated with only BUN and creatinine levels. In the early term group, total renal functions, access site functions, and BUN levels changed insignificantly. The treated sides' functions decreased (*P* = 0.02), the non-treated sides' functions increased (*P* = 0.02), and creatinine levels increased (*P* = 0.008) significantly. Also, in the late term group, the increase in the total functions and treated sides' functions was remarkable (resp. *P* = 0.005 and 0.04). 

## 4. Discussion

First of all, we should discuss the time of evaluation. When we looked at the literature, we saw that many writers had evaluated their patients in a limited time after surgery. Some of them did so within a few hours, some within a few days, and others prolonged evaluation for several months. We also recognized that the results differed from each other. Therefore, it could be said that evaluation time is important in determining the effects of PCNL on renal functions.

According to most of the literature on this issue, PCNL is a reliable and favorable surgical procedure. Most studies about the effect of PCNL on the kidney have a number of patients below 20 and focus on early period after surgery. Therefore, there can be statistical problems in their results. Additionally, evaluating PCNL in only the early term could be misleading as PCNL could be seen as harmful for kidney functions when the kidneys are responding to a surgical trauma [5]. Kawamura et al. stipulated 6 months after surgery for an exact assessment [4]. In the late term renal functions are preserved in a huge group of patients. When we examined our results month by month we saw that there was a significant increase after 6 months (*P* = 0.005). Therefore, we think Kawamura's claim is appropriate. In considering Handa's studies, we divided evaluating time into three: very early term (postoperative 5 hours), early term (5 hours–6 months), and late term (after 6th month).

Examining the effect of PCNL on a kidney is a complex issue. Evaluating this in vivo is very difficult in the presence of a healthy contralateral kidney. To overcome this problem, researchers performed PCNL on patients with solitary kidney. Resorlu et al. [6] performed PCNL on 16 patients with solitary kidney. After a year of followup, the glomerular filtration rate and creatinine levels showed healing. Liou and Streem [7] evaluated 83 patients with solitary kidney in whom ESWL, PCNL, or a combination of these was performed by measuring creatinine, blood pressure, and glomerular filtration rate levels before and after surgery. They were not able to obtain a significant change in the parameters and defined these therapy alternatives as equally reliable. With these results in a series with solitary kidney, PCNL's reliability was shown. However the small number of patients in the series could be seen as a limitation. Therefore, researchers performed simultaneous bilateral PCNL in patients with disease involving both kidneys. They could, therefore, achieve more case counts. 

Holman et al. [8] compared 150 patients who had undergone simultaneous bilateral PCNL with 300 patients who underwent unilateral PCNL by considering complications, success rates, and laboratory levels. As a result, they did not find any significant difference between the procedures in terms of these parameters. Handa et al. indicated that PCNL treatment of bilateral stones in one session was desirable because complications (functional or otherwise) did not appear to be significantly increased by this method of intervention [9]. 

Some researchers made their evaluation through histological assessment. Wilson et al. [10] compared pyelolithotomy (*n* = 3), nephrolithotomy (*n* = 4), PCNL (*n* = 5), and percutaneous nephrostomy placement by balloon dilatation (*n* = 5) with each other histologically before and after (postoperative 1st month) the surgery. After nephrectomy, at the 1st month after surgery, they found only minimal focal trauma to the access or entrance site. Clayman et al. [11]. researched the effects of 36 F and 24 F balloon dilatators on renal parenchyma at the same access site. Half of the access line was dilated with a 36 F balloon and the rest with a 24 F balloon. After 6 weeks, they extracted the sample kidneys of 12 pigs and evaluated the scars histologically. When they compared the surface area of the scars of both dilatation sites with the renal parenchyma, they could not find any significant difference between them. In addition, the total scarring to the renal surface was only at the rate of 0.15%. Handa et al. [5] compared balloon dilatation with Amplatz dilatations. They extracted the accessed kidneys of 15 pigs at the 4th postoperative hour and evaluated them histopathologically. At the access points, changes secondary to blunt trauma and ischemia were observed. There were corticomedullary parenchymal injuries along the access tracks. The diameter of access versus diameter of parenchymal injury proportions did not differ between the Amplatz and balloon dilatators. They determined that contralateral kidney functions deteriorated and believed that this resulted from the physiological response to surgical trauma. They stated that these negative findings were transient according to early data of another study they had made. The study showed them that this transient period lasted 3 days but that deficiencies in tubular functions could be permanent. They stated that an access does not only cause changes in the access site but that the whole kidney is also affected by an access, based on the renal vasoconstriction's magnitude that they had determined. Nazaroglu et al.'s [12] study supported this view. In these studies, renal vasoconstriction was determined by using Doppler ultrasonography in ESWL performed patients. In this way Handa showed that an area of 2–2.3 times bigger than the access track around the access site is affected based on the histological finding of the existence of damaged vessels in this area. Therefore, they supposed that damage to the kidney may be greater than previously thought. 

The effect of PCNL on kidney function was researched with radionuclide studies. Moskovitz et al. [3] evaluated their cases with single-photon emission computed tomography (SPECT) in terms of DMSA uptake. They considered uptake in each kidney and accessed poles and nonaccessed poles separately and also total renal uptake. They determined a significant decrease in the function of the accessed pole. The other parameters were not significantly affected in the early term. In another similar study, Ünsal et al. [13] compared percutaneous dilatation methods (balloon, Amplatz, and Alken). They found that they did not affect the renal functions at the end. Samad et al. [14] researched 60 renal units by using DMSA scan at the first postoperative month. They only found renal scars in 17% of cases. However, they mentioned that scar formation might be related with PCNL in only 5% of all cases. Dawaba et al. [15] studied DMSA and DTPA in 72 renal units. None of them had a renal scar. Ekelund et al. [16] determined that renal functions worsen scintigraphically on the postoperative 1st day but recover after 2 weeks. In the light of DMSA and GFR, Mayo et al. [17] claimed that PCNL protects the renal functions. 

In our study, there were no significant changes in the total renal functions scintigraphically. Although our postoperative creatinine levels show a similarity with those of Handa and Kuzgunbay, point to a deterioration in the very early term, the insignificance of DMSA values may be considered as the renal functions are preserved (Table 1). Our results showed that there is a significant decrease in renal functions in the accessed kidney (*P* = 0.003) (Table 2). As seen, this deterioration is significant for both early and late term patients. Besides, there is a significant increase in the nonaccessed side (*P* = 0.014). It is prominent especially in the early term (*P* = 0.01). It does not make sense to think both kidneys work separately. A deterioration in one kidney is compensated for by the other [18]. However, bilateral renal vasoconstriction has been shown after surgery in the very early hours after surgery [5, 12]. Mediators from damaged tissue may be responsible for the vasoconstriction in the kidney that has experienced trauma. However, we do not have enough knowledge about the contralateral kidney's functional changes. In scientific tradition, there are many reports about the compensation of the kidneys. The decrease in the accessed kidney's function, the increase in contralateral kidney's function, and no change in the total function in our study indicate the existence of this kind of compensation after PCNL access in the very early term. 

In Ünsal's study, there was no functional change in terms of access site. A point that Moskovitz and we agree on is a significant functional decrease in the access site (Table 3). Demographical or clinical factors do not affect this functional change; this means that this change is related to PCNL. In the light of Clayman et al.'s and Samad et al.'s studies [11, 14] we know that this functional decrease is transient and that there will be no significant scar in the kidney tissue.

Our results are similar to those of Moskovitz's study about the response of the accessed kidney's other nonaccessed poles to surgical trauma. According to our results it can be hypothesised that nonaccessed regions do not accompany contralateral kidney in compensation (*P* = 0.43). On the other hand, it whets our curiousity about how compensation occurs in those patients with solitary kidney or have undergone SBPCNL. Besides, the access site is not always the place where the stone is located. Therefore, the damages caused by access and stones are quite different. New studies are needed to enlighten these issues.

Although the results of some studies like Handa et al.'s [5] look like PCNL gives harm to the kidney tissue and functions, large studies like Holman et al.'s and Dawaba et al.'s [8, 15] tell us that PCNL is not harmful even in the early term. Besides, it improves renal functions. However, we have to consider the existence of a response to surgical trauma induced by PCNL. 

Tract and dilatation counts are also issues of PCNL that have been studied. Samad et al. and Eshghi et al. [14, 19] did not find any evidence against multiaccessing. Handa et al. [5] observed that the papilla on the access route was devastated. Therefore, they claimed that there may be papillary failure on pH regulation after multiaccesses or recurrent PCNL operations. However, they were not able to prove their thesis in the study that compared multi versus single access in 2009 [20].

In our study, 26 patients underwent single and 11 underwent multiaccess PCNL. These groups were compared with each other according to the functions of both kidneys, treated kidney, and non-treated kidney (Table 4). In the single access group the total function's increase was significant. On the other hand, the multiaccess group showed no significant changes in total function. However, the accessed kidney's function in the multiaccess group was significantly decreased. Although this suggests that multi access causes more damage to the kidney, the absence of a significant difference between the two groups at the end indicates that the success of multiaccess PCNL is not affected.

## 5. Conclusion

The time spans very early term, early term, and after 6 months that is, late term, are mentioned. It is a fact that there is a transient loss of kidney functions in the very early hours after surgery but after a while it recovers. PCNL does not cause any harm to the kidney locally or generally. In contrast, significant benefits can be seen after 6 months. Moreover, PCNL is still a reliable and favorable surgical procedure for the treatment of renal stones even if it is performed with the multiaccess technique.

## Figures and Tables

**Table 1:** The changes in total functions and BUN-creatinine levels before and after surgery are shown.

All patients (*n*) 37	Before PCNL	After PCNL	*P*
Total scintigraphic functions of kidneys	21.9 ± 11.7	22.7 ± 6.3	0.054
BUN	13.9 ± 3.5	13.0 ± 4.8	0.79
Creatinine	0.9 ± 0.2	1.0 ± 0.4	0.03

PCNL: percutaneous nephrolithotomy.

**Table 2:** Comparison of the functions of treated and nontreated sides' functions.

	Treated side	*P*	Nontreated side	*P*
Preoperative	45.8 ± 16.3	0.003	54.9 ± 16.9	0.014
Postoperative	43 ± 17.2		56.9 ± 17.2	
DMSA, before the 6th month				
Preoperative	44.4 ± 17.6	0.02	51.1 ± 18.3	0.01
Postoperative	41.1 ± 18.6		53.5 ± 18.8	
DMSA, after the 6th month				
Preoperative	48.8 ± 13.5	0.04	62.8 ± 10.05	0.42
Postoperative	47.08 ± 13.7		64.0 ± 10.7	

DMSA: Technetium-99m-Dimercaptosuccinic Acid.

**Table 3:** Changes in access site before and after surgery.

	Access site function (*n* = 51)	*P*
Preoperative DMSA	45.1 ± 16	0.041
Postoperative DMSA	42.7 ± 17.6	
Before the 6th month DMSA,		
Preoperative	45.0 ± 17.9	0.27
Postoperative	42.4 ± 19.9	
After the 6th month DMSA,		
Preoperative	50.4 ± 15.1	0.11
Postoperative	49.1 ± 14.7	

DMSA: Technetium-99m-Dimercaptosuccinic Acid.

**Table 4:** Comparison of single and multiaccess percutaneous nephrolithotomy.

	Single access		*P* value	Multiple access		*P* value
	Before surgery	After surgery		Before surgery	After surgery	
Total function	22.4 ± 13.3	22.7 ± 5.3	0.03	20.7 ± 7.2	22.5 ± 8.5	0.42
Treated side	48 ± 18	46.2 ± 18.5	0.06	40.6 ± 10.3	35.5 ± 10.8	0.01
Nontreated side	51.9 ± 18	53.7 ± 18.5	0.02	62 ± 11.5	64.4 ± 10.8	0.38
BUN	14.5 ± 3.5	13.5 ± 5.3	0.17	12.5 ± 3.04	11.6 ± 3.55	0.19
Creatinine	0.87 ± 0.17	1.0 ± 0.24	0.003	0.96 ± 0.26	1.0 ± 0.24	0.58
